# A Novel Strategy to Enhance the Photostability of InP/ZnSe/ZnS Quantum Dots with Zr Doping

**DOI:** 10.3390/nano12224044

**Published:** 2022-11-17

**Authors:** Xunqiang Cheng, Mingming Liu, Qinggang Zhang, Mengda He, Xinrong Liao, Qun Wan, Wenji Zhan, Long Kong, Liang Li

**Affiliations:** 1 School of Environment Science and Engineering, Shanghai Jiao Tong University, Shanghai 200240, China; 2 Macao Institute of Materials Science and Engineering (MIMSE), MUST-SUDA Joint Research Center for Advanced Functional Materials, Zhuhai MUST Science and Technology Research Institute, Macau University of Science and Technology, Taipa, Macao 999078, China

**Keywords:** InP/ZnSe/ZnS QDs, photostability, Zr doping

## Abstract

Plentiful research of InP semiconductor quantum dots (QDs) has been launched over the past few decades for their excellent photoluminescence properties and environmentally friendly characteristics in various applications. However, InP QDs show inferior photostability because they are extremely sensitive to the ambient environment. In this study, we propose a novel method to enhance the photostability of InP/ZnSe/ZnS QDs by doping zirconium into the ZnS layer. We certify that Zr can be oxidized to Zr oxides, which can prevent the QDs from suffering oxidation during light irradiation. The InP/ZnSe/ZnS:Zr QDs maintained 78% of the original photoluminescence quantum yields without significant photodegradation under the irradiation of LED light (450 nm, 3.0 W power intensity) for 14 h, while conventional InP/ZnSe/ZnS QDs dramatically decreased to 29%.

## 1. Introduction

Colloidal semiconductor quantum dots (QDs), which exhibit size-dependent emission color, and high photoluminescence quantum yields (PLQYs) [1,2,3], have been abundantly investigated in lighting, biological imaging, and other fields [4,5,6,7]. Among the QDs, inorganic metal chalcogenide QDs, such as CdS [8,9], CdSe [10,11], and PbS [12], have been extensively investigated during the past decade. However, despite the appealing properties, the intrinsic toxicity of cadmium and lead severely restricts their application, especially in view of environmental regulations and the restriction of hazardous substances [13,14]. 

Indium phosphide (InP) QDs are gaining increased attention and are regarded as the most potential alternatives to metal chalcogenide QDs because of their more environmentally friendly constituting elements [15,16]. Nevertheless, the studies of InP QDs are rather sparse in comparison with the cadmium chalcogenides, principally resulting from the difficulties of their synthesis [17]. More importantly, the photo-/chemical-stability of InP QDs is lower than Cd-based QDs and will inevitably be influenced by contact with air and moisture, which restrict their industrial and commercial applications [18]. In Kulakovich et al.’s work [19], they reported that the gold nanoparticles can not only improve the photostability of InP/ZnSe/ZnSeS/ZnS QDs, but also enhance the luminescence intensity, which is opposite from silver nanoparticles. In addition, temperature also has a great impact on the stability of InP-based QDs. Related studies have reported that the emission intensity of InP QDs decreases with the increase of temperature [20], and temperature can also cause a change of the position and width of the first exciton absorption peak and PL peak of QDs [21,22]. Therefore, effective strategies should be proposed to enhance the stability of InP QDs.

The constitution of core/shell structure QDs through coating with semiconductor shell layers is usually deemed to be an effective approach in improving the photostability of QDs [23,24]. The shell layers provide a wide bandgap to efficiently confine the excitons of the core and also work as the physical barrier to prevent the erosion of water and moisture [25]. Unfortunately, previous works have reported that the quantum efficiency of InP/ZnSe/ZnS QDs decreases significantly when the shell thickness reaches a certain level due to the crystal defects [26,27], which can’t be offset by the inconspicuous increase of photostability. As a result, in the current synthesis schemes, most researchers coat the InP core with a relatively thick shell. Therefore, uncovering methods to further improve the photostability of InP/ZnSe/ZnS QDs based on appropriately increasing the shell thickness has become a troublesome problem in this field.

Apart from the construct of type-Ⅰ core/shell structure, the metal elements doping strategy has been proven to effectively enhance the photostability of QDs [28,29]. Koh et al. found that Al-doped InP/ZnSeS/ZnS QDs can retain 81.6% of original QYs under 180 °C for 4 h, but they did not study the photostability of QDs [30]. Our previous work reported that CdSe/CdS QDs doping with aluminum (Al) metal can well maintain their fluorescence without sacrificing the quantum efficiency and the photostability under ultraviolet light [11,31]. These metallic elements are incorporated into the QDs and form metal oxides, which can enhance the stability of QDs. Zr, as a doped element, is easy to form zirconium oxides with a Gibbs free energy for the formation of ZrO_2_ (−1042.8 kJ mol^−1^) [32] in the standard temperature and pressure, which is similar to aluminum and can perform a wonderful effect on hindering corrosion from water and oxygen. To the best of our knowledge, no related literature has been reported on the Zr-doped InP/ZnSe/ZnS QDs and photostability along with the mechanism.

**Figure 1:**
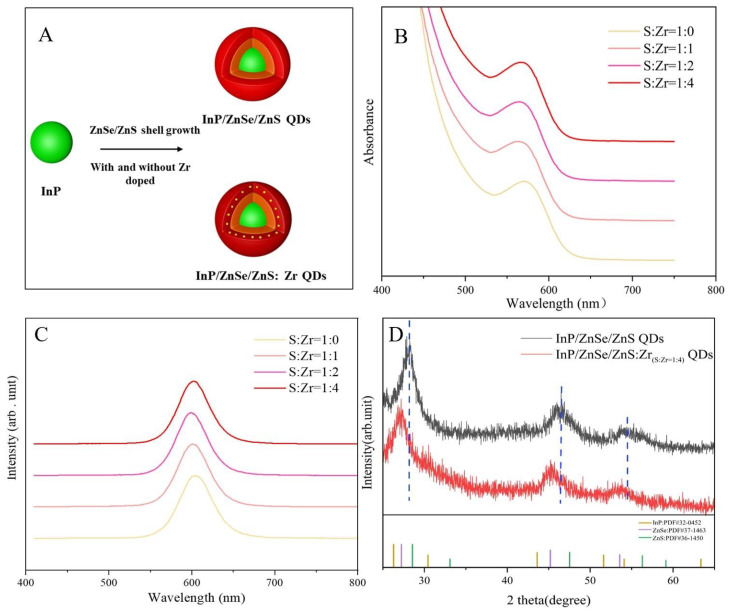
(**A**) Schematic of the synthesis of QDs. (**B**) UV-vis absorption and (**C**) PL spectra of QDs with different S/Zr ratios. (**D**) XRD patterns of QDs.

In this study, we propose a novel synthesis method to coat Zr-doped ZnS layers over the InP/ZnSe QDs using a Zirconium acetate (Zr(Ac)_4_) solution (dissolved in acetic acid) to improve their photostability. A schematic (Figure 1A) is proposed to describe the synthesis of InP/ZnSe/ZnS:Zr QDs. As we know, with the increase of shell thickness, the photostability of InP QDs will be enhanced. However, the incorporation of Zr into the ZnS layers remarkably strengthens the photostability of QDs compared with the counterpart of InP/ZnSe/ZnS QDs without Zr doping. Under the illumination of a blue LED, the doped Zr gradually forms zirconium oxides and then works as a protective layer to prevent the oxidation of ZnS. What makes this research more impressive is that the doping of Zr not only does not alter the optical properties of the QDs, but also offers ultra-stable photoluminescence, even under exposure to LED with high power.

## 2. Materials and Methods

### 2.1. Chemicals

Indium (III) acetate [In(Ac)_3_, ≥99.99%], myristic acid (MA, ≥99%), zinc (II) stearate [Zn(St)_2_, >99%], tri-η-octyl phosphine (TOP, ≥97%), 1-octadecene (ODE, technical grade), selenium powder (Se, ≥99.99%), sulfur powder (S, ≥99.98%), acetic acid solution (HAc, ≥99%), and Zirconium acetate solution in dilute acetic acid [Zr(Ac)_4_, Zr: 15.0–17.0% gravimetric] were purchased from Sigma-Aldrich. Tris(trimethylsilyl) phosphine ((TMS)_3_P, >98%, 10 wt.% in hexane) were purchased from Strem Chemicals. Toluene (anhydrous, ≥99.8%), acetone (anhydrous, ≥99.8%), and ethanol (anhydrous, ≥99.8%) were purchased from General-reagent.

### 2.2. Preparation of TOP-Se and TOP-S

An amount of 40.0 mmol of Se was dissolved in 20.0 mL of TOP and stirred at room temperature. After the solution was completely clear and transparent, 60.0 mL of ODE was added and stirred to mix them thoroughly. The preparation of 0.5 M TOP-S followed the same method.

### 2.3. Synthesis of InP Core QDs

A total of 0.6 mmol of In(Ac)_3_, 0.2 mmol of MA, and 6.0 mL of ODE were added into a 50 mL round-bottom four-neck flask equipped with a reflux condenser and a thermocouple probe under an air-free condition. The container was evacuated and charged with argon several times to isolate oxygen thoroughly at room temperature. The flask was heated and stirred for 1 h at 130 °C with a heating mantle and under a near-vacuumed condition. The argon was purged into the reaction system and the reaction temperature was then raised to 300 °C. The mixture of 0.4 mL of (TMS)_3_P and 1.0 mL of TOP was injected into the flask rapidly and then kept at a temperature of 290 °C for 5 min for the growth of InP nanocrystals.

### 2.4. Synthesis of InP/ZnSe QDs

After the growth of InP QDs, a mixture of 0.1 mmol of Zn(St)_2_ and 0.1 mmol of TOP-Se diluted with 2.0 mL of ODE was quickly added to the reaction system and kept at 300 °C for 30 min to form a thin ZnSe layer. To coat with the ZnSe, 0.4 mmol of Zn(St)_2_ dissolved with 3.0 mL of ODE was drawn up with a syringe. After 10 min, 0.4 mmol of TOP-Se was injected into the flask using a syringe pump at a rate of 1.6 mL/h. The whole system stayed at 300 °C for another 30 min. We repeated these steps once more for the formation of a thick ZnSe layer.

### 2.5. Synthesis of Different Kinds of InP/ZnSe/ZnS QDs

To synthesize the InP/ZnSe/ZnS-thin QDs, 0.8 mmol of Zn(St)_2_ and 3.0 mL of ODE were blended and then injected into the flask. The mixture was heated at 300 °C within 10 min, following the dropwise injection of 0.8 mmol of TOP-S at a rate of 3.2 mL/h. The system stayed at 300 °C for another 30 min and then cooled to room temperature.

To synthesize the InP/ZnSe/ZnS-thick QDs (InP/ZnSe/ZnS QDs with a thicker ZnS layer), all the experimental schemes followed the same procedures of InP/ZnSe/ZnS-thin QDs and the steps for coating the ZnS layer were twice.

To synthesize the InP/ZnSe/ZnS QDs (InP/ZnSe/ZnS-thick QDs with acetic acid treated), 1.2 mL of HAc was introduced during the growth of the thicker ZnS layer.

To synthesize the InP/ZnSe/ZnS:Zr QDs (InP/ZnSe/ZnS QDs with Zr doped) with different Zr concentrations, a Zr(Ac)_4_ solution was injected into the flask together with the TOP-S solution.

### 2.6. Purification of InP/ZnSe/ZnS QDs

The crude QDs were extracted three times with 50 mL of ethanol at 60 °C. After the extraction steps, the QDs were dispersed in 10 mL of toluene and then mixed with 45 mL of acetone. After centrifugation for 5 min at 10,000 rpm, acetone was added to precipitate the QDs, followed by centrifugation and decantation. The supernatant was discarded, and the precipitate was redispersed in 10 mL toluene. The mixture was centrifuged for 5 min at 8000 rpm and then reserved supernatant. This washing process was executed twice more to remove all the by-products and unreacted reagents and the QDs were dispersed in toluene for further use.

### 2.7. Characterization

UV-vis absorption spectra and Photoluminescence (PL) spectra were recorded by a Cary-60 UV-vis spectrophotometer and an F-380 fluorescence spectrometer (Tianjin Gangdong Sci. & Tech. Development Co., Ltd., Tianjin, China), respectively. The high-resolution transmission electron microscope (HRTEM) images and energy dispersive X-ray spectroscopy (EDX) analysis were characterized using the FEI Talos F200X TEM instruments operated at an accelerating voltage of 200 kV. X-ray powder diffraction (XRD) patterns of samples were performed on a Bruker D8 Advance X-ray Diffractometer at 40 kV and 40 mA using Cu Kαradiation (λ = 1.5406 Å). The photoluminescence quantum yield was obtained using an integrating sphere with a Hamamatsu Quantaurus-QY Absolute PL quantum yield spectrometer (Model: C11347-11). X-ray photoelectron spectroscopy (XPS) analysis was recorded by a Kratos Axis Ultra-DLD spectrometer, and all the spectra were calibrated to the C1s peak at 284.8 eV.

### 2.8. Photostability Test

The QDs with the same volume of 20 mL were kept in sealed glass bottles with transparent bottoms and the absorbances of these QDs at 470 nm were 0.62. The UV-vis absorption and PL spectra of these QDs were measured and recorded at each periodic interval under illumination with an LED light at 450 nm and 3.0 W power intensity.

## 3. Results and Discussion

Firstly, the core/shell structure InP/ZnSe/ZnS QDs with different thicknesses of ZnS shell layer, labeled as InP/ZnSe/ZnS-thin QDs and InP/ZnSe/ZnS-thick QDs, were synthesized to explore the influence of shell thickness on their photostability. Figure S1 shows their morphology features, and Figure S2 shows the photostability of these QDs. Obviously, the InP/ZnSe/ZnS QDs with thicker ZnS layers exhibited better photostability. The InP/ZnSe/ZnS-thick QDs maintained 50% of initial fluorescence intensity after the irradiation for 11 h, but the thin ones only maintained intensity for 7 h. When the illumination time reached 30 h, the PLQYs of these QDs decreased significantly, only maintaining 10% and 19% of their original intensity, which is far from commercial applications, demonstrating that increasing the thickness of the ZnS layer only slightly improves the photostability of InP/ZnSe/ZnS QDs. This phenomenon is quite different from the observation from the coating of CdSe QDs, in which continuously increasing the shell thickness beyond 10 nm generally could obtain giant dots, which was proved as ultra-stable QDs. However, in the coating of InP QDs, we found that further increasing the thickness of ZnS usually deteriorated the properties of the InP core/shell samples, such as dropped PLQY and worsened size distribution.

To further improve the photostability of InP/ZnSe/ZnS QDs with a relatively thick ZnS layer, Zr was introduced during the growth of ZnS layers to form Zr-doped InP/ZnSe/ZnS (labeled as InP/ZnSe/ZnS:Zr) QDs. Notably, the Zr(Ac)_4_ solution utilized in this work contained acetic acid, hence we synthesized the InP/ZnSe/ZnS-thick QDs with equal acetic acid (labeled as InP/ZnSe/ZnS QDs) as the comparison. Therefore, InP/ZnSe/ZnS QDs and InP/ZnSe/ZnS:Zr QDs with different doping ratios were synthesized to verify the effect of the Zr doped and simultaneously explored the influence of the amount of Zr doped on the photostability of InP/ZnSe/ZnS:Zr QDs.

**Figure 2:**
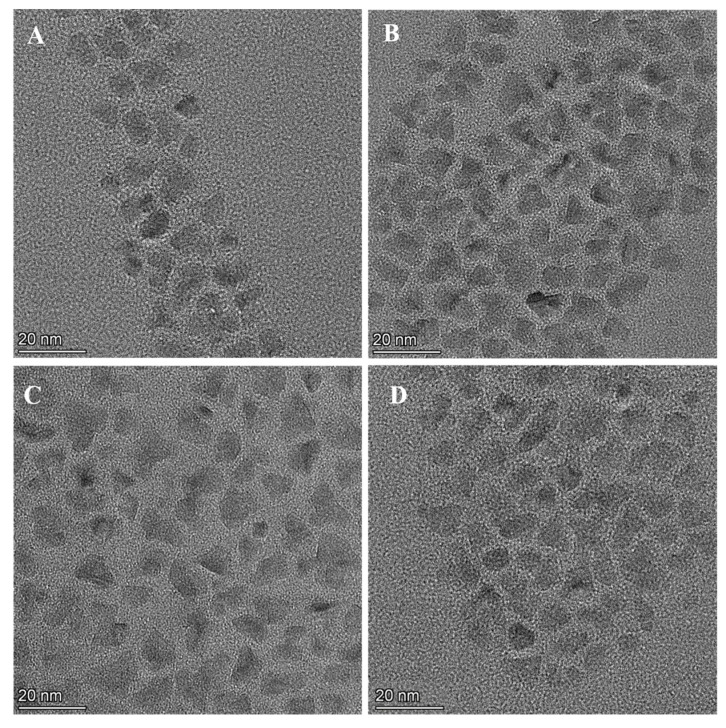
TEM images of (**A**) InP/ZnSe/ZnS and (**B**) InP/ZnSe/ZnS:Zr (with the ratio of S/Zr = 1:4) QDs before irradiation of LED. TEM images of (**C**) InP/ZnSe/ZnS and (**D**) InP/ZnSe/ZnS:Zr (with the ratio of S/Zr = 1:4) QDs after irradiation of LED.

Figure 1 shows the schematic of the synthesis of InP/ZnSe/ZnS and InP/ZnSe/ZnS:Zr QDs (A), UV-vis absorption (B), and PL spectra (C) of InP/ZnSe/ZnS and InP/ZnSe/ZnS:Zr QDs. The PLQYs of undoped and Zr-doped InP/ZnSe/ZnS QDs are 45% and 47%. There is a slight difference in the optical spectra of InP/ZnSe/ZnS and InP/ZnSe/ZnS:Zr QDs and the PL peaks are at 604 nm (InP/ZnSe/ZnS QDs) and 602 nm (InP/ZnSe/ZnS:Zr (S/Zr = 1:4) (Figure 1B,C, Table S1). By comparing the TEM images of InP/ZnSe/ZnS and InP/ZnSe/ZnS:Zr (with the ratio of S/Zr = 1:4) QDs (Figure 2A,B), we found that the two samples exhibit similar particle sizes, which are both around 7.5 nm (Figure S3). The interplanar spacing of InP/ZnSe/ZnS QDs is 0.33 nm, corresponding to the (100) crystallographic plane of ZnS, while the crystal plane spacing of InP/ZnSe/ZnS:Zr QDs is 0.34 nm, slightly larger than that of undoped QDs (Figure S4). The XRD pattern of InP/ZnSe/ZnS and InP/ZnSe/ZnS:Zr QDs (Figure 1D) both exhibited three preferential peaks and these peaks of InP/ZnSe/ZnS:Zr QDs obviously shifted to smaller angles, which is attributed to the partial substitution of Zn atoms by larger Zr atoms in the ZnS lattice, resulting in the lattice expansion of the ZnS layer. These results demonstrate that the incorporation of Zr has no distinct effect on the morphology, size distribution, and structure of InP/ZnSe/ZnS QDs. In addition, the EDX was used to clarify the amount of Zr incorporated into the ZnS layer (Table S2). All samples were cleaned repeatedly with toluene and acetone. When the theoretical doping dose of Zr (S/Zr) is 1:4, the real ratio of Zn to Zr in the final sample is 2.20. Figures S5 and S6 show the elemental mapping of the In, P, Zn, Se, S, and Zr of InP/ZnSe/ZnS and InP/ZnSe/ZnS:Zr QDs respectively, from which it can clearly be observed that the distribution of various elements highly matches the QDs.

**Figure 3:**
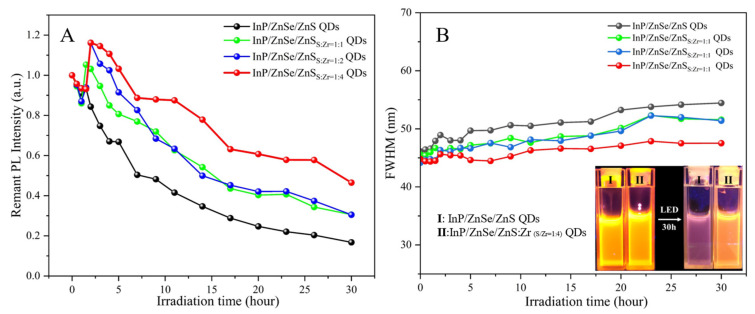
(**A**) The PL intensity and (**B**) FWHM of InP/ZnSe/ZnS and InP/ZnSe/ZnS:Zr QDs with different Zr concentrations under the irradiation of LED at 450 nm and 3.0 W over time.

The photostability of InP/ZnSe/ZnS and InP/ZnSe/ZnS:Zr QDs with different Zr doped concentrations were demonstrated by continuous illumination under an LED light and the fluorescence intensities of these QDs changed with time, as shown in Figure 3A. The fluorescence intensity of InP/ZnSe/ZnS QDs decreased to 50% of the initial intensity after 7 h. All three InP/ZnSe/ZnS:Zr samples kept much higher percentages of their original PLQYs, which indicates the photostability of InP/ZnSe/ZnS:Zr QDs were significantly improved with the incorporation of Zr. The photostability of these QDs with doped Zr exhibited an obviously increasing trend with the rising doping dose of Zr. Within the scope of our experiment, the InP/ZnSe/ZnS:Zr QDs presented the best photostability when the theoretical doping ratio of S/Zr is 1:4, which could retain 58% of their initial PL intensity after 26 h of irradiation. Combined with the EDX analysis of the actual Zr concentration incorporated into the ZnS layer, we can obviously conclude that the doped Zr can significantly improve the photostability of InP/ZnSe/ZnS QDs. Interestingly, we also observed that the PL intensity of InP/ZnSe/ZnS:Zr QDs decreased significantly within the first 1.5 h of irradiation and then increased sharply, even surpassing the original PL intensity due to the photo-brightening effects [33,34]. However, the initial drop of PL is caused by the doped Zr and did not form an effective Zr oxides self-passivation layer at the initial stage within 1.5 h, which is similar to what happened on the Al-doped CdSe/CdS QDs. After several hours of illumination of blue light, the Zr atoms gradually formed a layer of Zr oxides, which covered the inside InP/ZnSe/ZnS QDs and effectively prohibited the further photoinduced degradation of QDs. By comparing TEM images (Figure 2) of undoped and doped InP/ZnSe/ZnS QDs before and after irradiation, we find that the morphology of these two QDs changed mildly. As a testing of concept, the InP/ZnSe/ZnS:Zr QDs were utilized for the reduction of Cr (Ⅵ) under simulated sunlight irradiation (Figure S7). Through monitoring the FWHM of these QDs within the irradiation process, it was indicated that the Zr-doped InP/ZnSe/ZnS QDs maintained a much better size distribution and the original PL properties (Figure 3B) than that of undoped one, which also proves their better colloidal stability during the irradiation process than that of the undoped sample.

**Figure 4:**
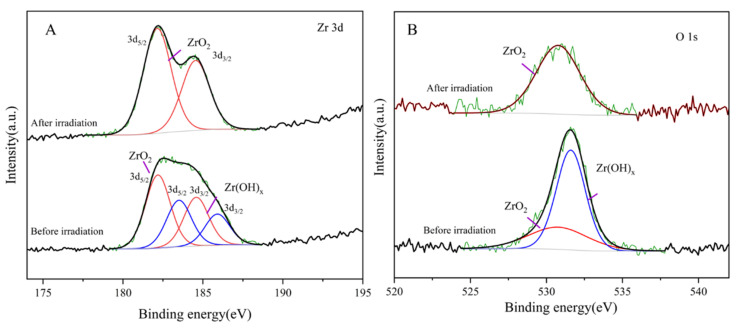
XPS of the (**A**) Zr3d region and (**B**) O1s region of InP/ZnSe/ZnS:Zr (S/Zr = 1:4) QDs before and after light irradiation.

To demonstrate the chemical states and the generation of the protective layer on QDs, the XPS analysis was conducted. As shown in Figure 4A, the Zr 3d XPS spectra of InP/ZnSe/ZnS:Zr QDs were checked. The Zr 3d 5/2 and Zr 3d 3/2 peaks appeared at 182.2 eV and 184.6 eV assigned to Zr-O [35] before irradiation, and the peaks at 184.4 eV and 186.8 eV were attributed to the Zr-OH [36], respectively. This indicates that partial oxidation of Zr occured before illumination, which may occur during the cleaning process. However, after illumination, there was only Zr 3d 5/2 peak and Zr 3d 3/2 peak for Zr-O, and the metal hydroxides disappeared completely. This indicates that Zr incorporated into the QDs is transformed from the Zr-OH state to the more stable Zr-O during the illumination process, which can protect the InP/ZnSe/ZnS QDs from erosion. To further explore the effect of the illumination process on the QDs, we also analyzed the XPS data of O1s. It can be seen from Figure 4B that most of the Zr in the doped QDs was in the form of hydroxide (531.7 eV) [37] and only a small amount of Zr was in the form of Zr-O (530.7 eV) [38] before irradiation. The peak position of O1s significantly turned to Zr-O after illumination, which strongly supports the conclusion mentioned above. The doped Zr, with a lower Gibbs free energy for the formation of ZrO_2_ (−1042.8 kJ mol^−1^) [32] as compared to that of ZnO (−320.5 kJ mol^−1^) [39], is preferential to transform into more stable Zr oxides after irradiation to isolate water and oxygen, which make the InP/ZnSeS/ZnS:Zr QDs exhibit good photostability.

**Scheme 1:**
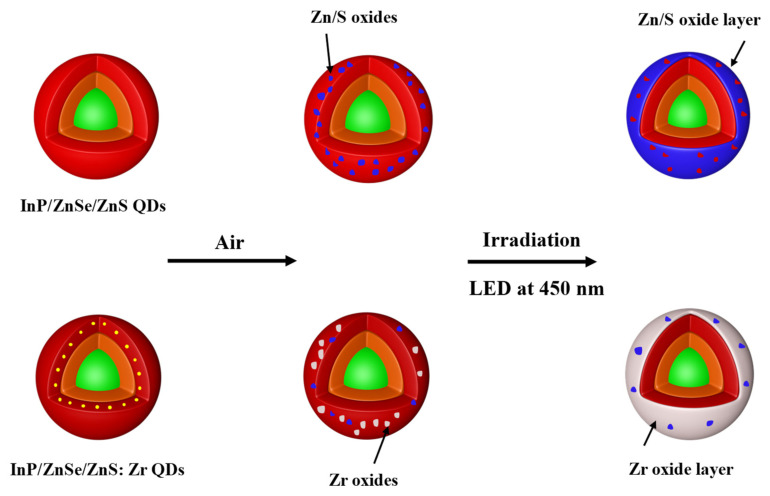
Schematic description of the degradation of InP/ZnSe/ZnS QDs and the tolerance of InP/ZnSeS/ZnS:Zr QDs under the irradiation of LED.

Scheme 1 shows a simple model to describe the process related to InP/ZnSe/ZnS:Z r QDs. When the QDs are exposed to an ambient environment, Zr is preferentially oxidized to Zr oxide in the ZnS layer, which plays a better role in isolating water and oxygen and can effectively prevent the oxidation of ZnS. However, in the InP/ZnSe/ZnS QDs without Zr, the ZnS layer is damaged, which leads to further oxidation of InP/ZnSe/ZnS QDs.

## 4. Conclusions

In summary, we developed a Zr doping strategy to enhance the photostability of InP/ZnSe/ZnS QDs. Based on coating a thick ZnS layer, the incorporation of Zr into the ZnS layer could make the InP/ZnSe/ZnS:Zr QDs maintain 58% of the original fluorescence intensity after 26 hours LED irradiation, while InP/ZnSe/ZnS QDs lost 80% of their PLQYs. Within the scope of our experiments, we verified three theoretical doped ratios of Zr and found that the photostability of InP/ZnSe/ZnS:Zr QDs improved with the increase of Zr concentration. After the photostability test, InP/ZnSe/ZnS:Zr QDs maintained their original morphology and had not been observably oxidized. Based on the XPS spectra of InP/ZnSe/ZnS:Zr QDs before and after irradiation, we further investigated the chemical transformation of Zr from Zr-OH to Zr-O during irradiation. The formed Zr oxide worked as a protection layer to prevent further oxidation of InP/ZnSe/ZnS:Zr QDs, thus greatly improving photostability. In addition, the physical characteristics of Zr itself indicate it has excellent high-temperature resistance and is often used as a refractory, therefore it also provides a consequential research direction in the improvement of the thermal stability of InP/ZnSe/ZnS QDs.

## Data Availability

Not applicable.

## Supplementary Materials

The following supporting information can be downloaded at: https://www.mdpi.com/article/10.3390/nano12224044/s1, Figure S1: TEM images of (A) InP/ZnSe/ZnS-thin and (B) InP/ZnSe/ZnS-thick QDs; Figure S2: The PL intensity of InP/ZnSe/ZnS-thin QDs and InP/ZnSe/ZnS-thick QDs under the irradiation of LED at 450 nm and 3.0 W over time; Figure S3: The size distribution of InP/ZnSe/ZnS and InP/ZnSe/ZnS:Zr (S/Zr=1:4) QDs; Figure S4: HRTEM images and lattice spacing of (A) InP/ZnSe/ZnS and (B) InP/ZnSe/ZnS:Zr (S/Zr = 1:4) QDs; Figure S5: HAADF-STEM image and elemental maps of the In, P, Zn, Se, and S of InP/ZnSe/ZnS QDs; Figure S6: HAADF-STEM image and elemental maps of the In, P, Zn, Se, S, and Zr of InP/ZnSe/ZnS:Zr QDs; Figure S7: The reduction of Cr (Ⅵ) with InP/ZnSe/ZnS:Zr QDs treated under simulated sunlight irradiation; Table S1: The position of PL peak and FWHM of InP/ZnSe/ZnS and InP/ZnSe/ZnS:Zr (with different Zr concentrations) QDs; Table S2: The EDX results of InP/ZnSe/ZnS:Zr QDs with the theoretical S: Zr molar ratios equaling 1:4.
