# Supplementary material for: A Novel Strategy to Enhance the Photostability of InP/ZnSe/ZnS Quantum Dots with Zr Doping
Source: Nanomaterials (Basel). 2022 Nov 17;12(22):4044. doi: 10.3390/nano12224044 (PMC9698936; doi:10.3390/nano12224044)
Supplement: Supplementary file 1 [file nanomaterials-12-04044-s001.zip › nanomaterials-2009220-supplementary.pdf]

A novel strategy to enhance the photostability of InP/ZnSe/ZnS Quantum Dots with Zr doping

Xunqiang Cheng [1], Mingming Liu [1], Qinggang Zhang [1], Mengda He [1], Xinrong Liao [1], Qun Wan [1], Wenji Zhan [1], Long Kong [1,*], and Liang Li [1,2]

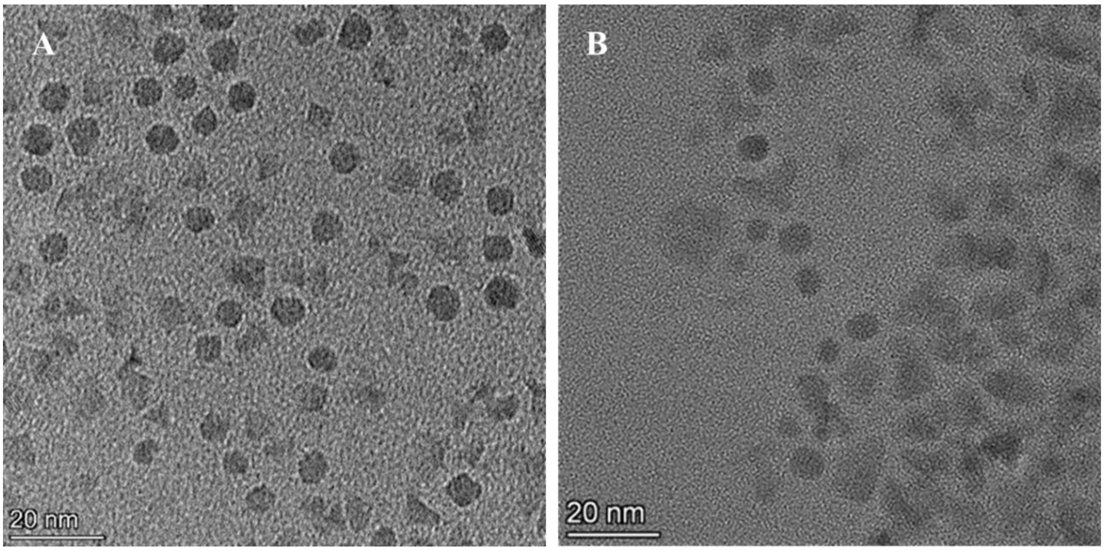

Figure S1. TEM images of (**A**) InP/ZnSe/ZnS-thin and (**B**) InP/ZnSe/ZnS-thick QDs.

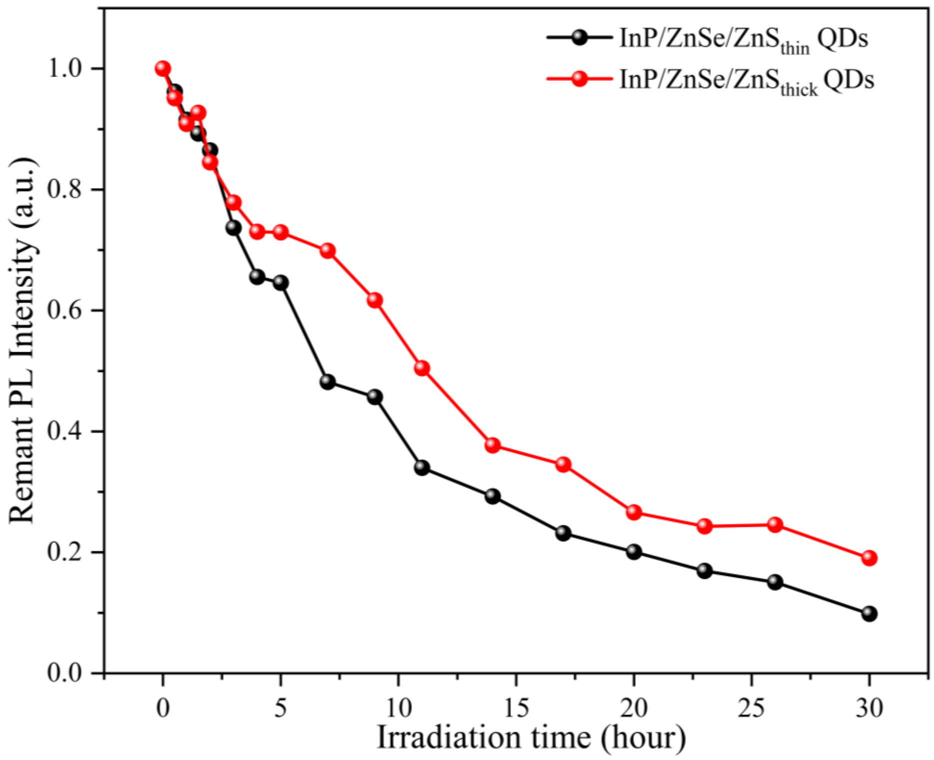

Figure S2. The PL intensity of InP/ZnSe/ZnS-thin QDs and InP/ZnSe/ZnS-thick QDs under the irradiation of LED at 450 nm and 3.0 W over time.

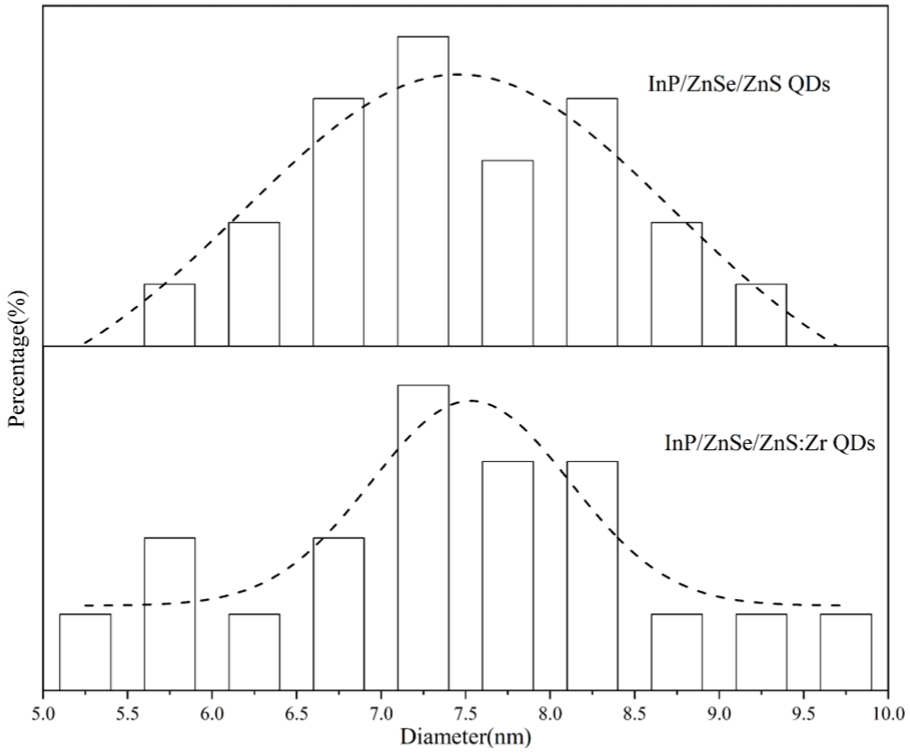

Figure S3. The size distribution of InP/ZnSe/ZnS and InP/ZnSe/ZnS:Zr (S/Zr=1:4) QDs.

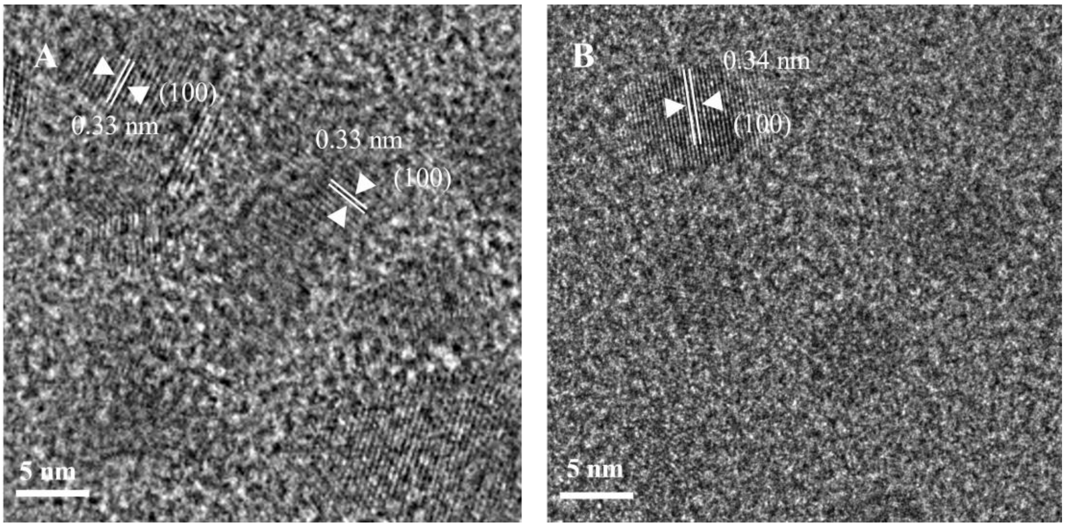

Figure S4. HRTEM images and lattice spacing of (**A**) InP/ZnSe/ZnS and (**B**) InP/ZnSe/ZnS:Zr (S/Zr=1:4) QDs.

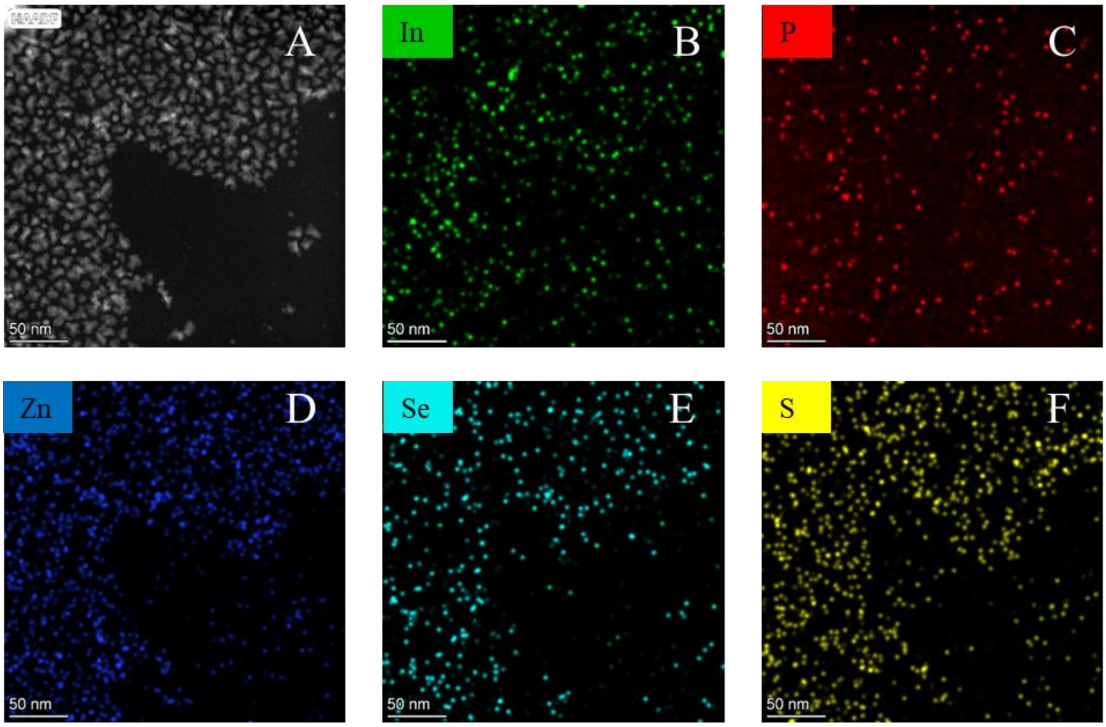

Figure S5. HAADF-STEM image and elemental maps of the In, P, Zn, Se, and S of InP/ZnSe/ZnS QDs.

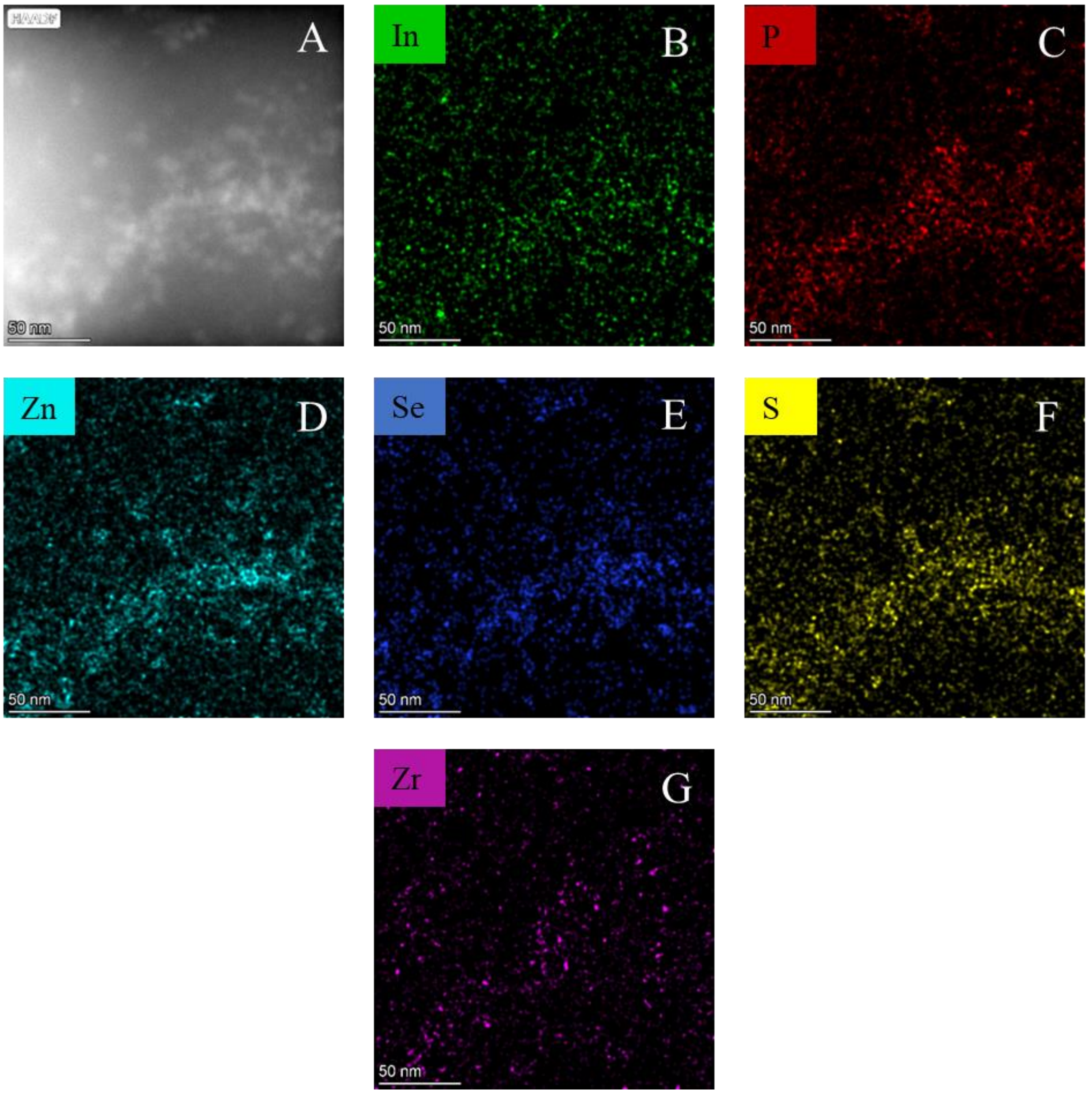

Figure S6. HAADF-STEM image and elemental maps of the In, P, Zn, Se, S, and Zr of InP/ZnSe/ZnS:Zr (S/Zr=1:4) QDs.

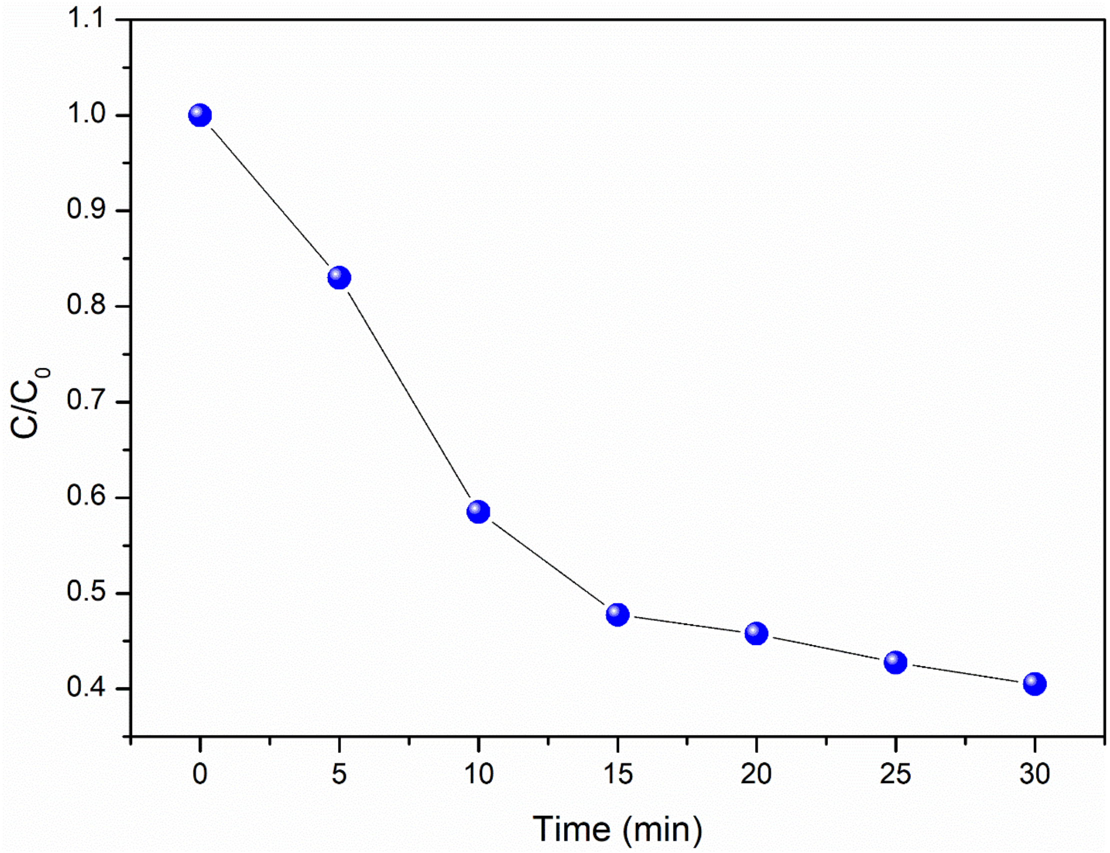

Figure S7. The reduction of Cr (VI) with InP/ZnSe/ZnS:Zr QDs treated under simulated sunlight irradiation.

Table S1: The position of PL peak and FWHM of InP/ZnSe/ZnS and InP/ZnSe/ZnS:Zr (with different Zr concentrations) QDs.

**S/Zr**	**Position of PL peak (nm)**	**FWHM (nm)**
1:0	604	45.9
1:1	601	44.2
1:2	600	44.5
1:4	602	44.7

Table S2: The EDX results of InP/ZnSe/ZnS:Zr QDs with the theoretical S: Zr molar ratios equaling 1:4.

Sample	Zn fraction (%)	Zr fraction (%)	Zn/Zr ratio
Sample 1	39.23	18.25	2.15
Sample 1	38.70	18.57	2.08
Sample 1	35.41	14.68	2.41
Average	37.78	17.17	2.20
